# Spatial Heterogeneity of Intratumoral Microbiota: A New Frontier in Cancer Immunotherapy Resistance

**DOI:** 10.3390/biomedicines13051261

**Published:** 2025-05-21

**Authors:** Qiwen Tan, Xiongjing Cao, Falong Zou, Hanwenchen Wang, Lijuan Xiong, Shenghe Deng

**Affiliations:** 1 Department of Infectious Disease, Union Hospital, Tongji Medical College, Huazhong University of Science and Technology, Wuhan 430022, China; 15703054626@163.com; 2 Department of Nosocomial Infection Management, Union Hospital, Tongji Medical College, Huazhong University of Science and Technology, Wuhan 430022, China; 13297033512@163.com; 3 Department of Gastrointestinal Surgery, Union Hospital, Tongji Medical College, Huazhong University of Science and Technology, Wuhan 430022, China; zoufalong@163.com (F.Z.); m202476342@hust.edu.cn (H.W.); 4 Center for Liver Transplantation, Union Hospital, Tongji Medical College, Huazhong University of Science and Technology, Wuhan 430022, China

**Keywords:** intratumoral microbiota, immune microenvironment, spatial heterogeneity, immunotherapy, resistance

## Abstract

The intratumoral microbiota, as an important component of the tumor microenvironment, is increasingly recognized as a key factor in regulating responses to cancer immunotherapy. Recent studies have revealed that the intratumoral microbiota is not uniformly distributed but instead exhibits significant spatial heterogeneity, with its distribution patterns influenced by factors such as tumor anatomy, local immune status, and therapeutic interventions. This spatial heterogeneity not only alters the interactions between microbes and the host immune system but may also reshape the immunogenic and immunosuppressive landscapes of tumors. The enrichment or depletion of microbiota in different tumor regions can influence immune cell infiltration patterns, metabolic pathway activities, and immune checkpoint molecule expression, thereby driving the development of resistance to immunotherapy. Moreover, certain bacterial metabolites form concentration gradients between the tumor core and margins, thereby regulating immune cell function. Therefore, understanding and manipulating the spatial distribution of intratumoral microbiota, particularly in resistant patients, holds promise for developing new strategies to overcome immunotherapy resistance. In the future, precise modulation strategies targeting microbial spatial heterogeneity, such as engineered bacterial vectors, probiotic combinations, and phage therapy, may open new avenues for immunotherapy.

## 1. Introduction

Tumors have long been considered sterile, with the tumor microenvironment (TME) thought to consist mainly of tumor cells, immune cells, blood vessels, and other components [1]. However, recent studies have challenged this traditional view by revealing the presence of intratumoral microbiota and their critical roles in tumor initiation and progression [2]. In the 19th century, Koch and Pasteur were among the first to observe bacteria within tumors [3], indicating that intratumoral microbial communities not only are specific but also profoundly influence tumor immune responses. Through interactions with immune cells, these microorganisms may either stimulate immune responses or foster tumor growth by exerting immunosuppressive effects [4]. The presence of intratumoral microbiota as a key part of the tumor microenvironment has driven new approaches in cancer immunotherapy. Although immune checkpoint inhibitors, such as PD-1/PD-L1 antibodies, have achieved groundbreaking progress in various cancers, the overall response rates to immunotherapy remain low, with clinical response rates below 30% [5]. The intratumoral microbiota can affect the outcomes of immunotherapy by modulating immune cell infiltration and function [6]. Thus, targeting the intratumoral microbiota could offer a promising approach to overcoming resistance to immunotherapy.

In recent years, research into the spatial heterogeneity of intratumoral microbiota has been increasingly recognized. Different regions within the tumor microenvironment may harbor distinct microbial communities, and this spatial distribution may play a critical role in responses to immunotherapy [7,8]. The spatial heterogeneity of microbiota offers new insights into the differential responses to tumor immunotherapy and highlights the importance of regulating the local microenvironment [9]. Consequently, future research should aim to manipulate the microbiota’s composition and spatial distribution to enhance the efficacy of immunotherapy. By thoroughly elucidating the interactions between intratumoral microbiota and the immune system, it may be possible to pioneer new directions for cancer immunotherapy and advance more individualized and precise therapeutic strategies.

## 2. Composition and Sources of Intratumoral Microbiota

**Figure 1:**
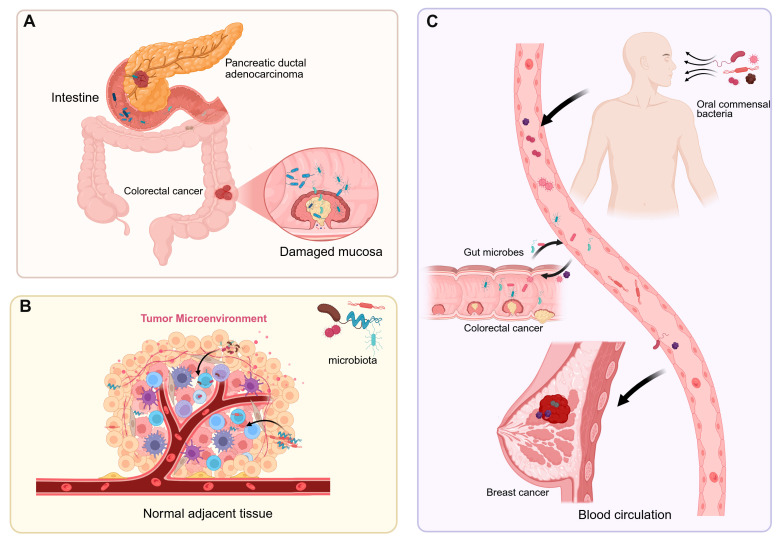
The sources of intratumoral microbiota. (**A**) Gut microbes migrate to tumor tissues through a compromised intestinal barrier, while bacteria in pancreatic ductal adenocarcinoma enter the pancreas via the pancreatic duct. (**B**) Normal adjacent tissues are one of the potential sources of intratumoral microbiota. (**C**) Intratumoral microbes enter tumor sites through the bloodstream from the oral cavity, intestines, tumors, and other locations.

Intratumoral microbiota interact with host cells and the tumor microenvironment (TME), thereby influencing tumor initiation, progression, and therapeutic responses [10]. The composition and origin of intratumoral microbiota have become a research hotspot at the intersection of tumor biology and microbiology [11]. The dominant microbial species vary among different cancer types, and their abundance is closely associated with the tumor microenvironment. The origins of intratumoral microbiota are not fully understood, but current research suggests they may enter tumor tissues through three main pathways (Figure 1). First is the migration from disrupted mucosal barriers: Gut microbiota, for instance, may breach a damaged intestinal barrier to reach tumor tissues [12]. Skin microbes adjacent to breast cancer could invade via mammary ducts [13]. In pancreatic ductal adenocarcinoma (PDAC), bacteria may transfer from the injured intestinal mucosa through pancreatic ducts, reshaping the TME and triggering both innate and adaptive immune suppression [14]. Second is migration from neighboring tissues or organs: Microbes have been found even in tumors originating from non-mucosal sites, indicating alternative sources for intratumoral microorganisms [15]. Nejman et al. discovered that microbial communities in breast and lung cancers shared similarities with adjacent normal tissues, implying that nearby healthy tissues could serve as a source of tumor-associated microbiota [11]. Additionally, the majority of bacteria detected in tumor tissues were found intracellularly, mainly within CD45+ immune cells, indicating that host cells may transport bacteria from nearby normal tissues to tumors [16,17]. Third is dissemination via blood circulation: Gut microbes may translocate into the bloodstream through the portal vein system and establish colonies within tumor tissues under certain conditions [18]. Oral commensal bacteria, such as *Fusobacterium nucleatum*, can reach tumors like breast cancer and colorectal cancer through the bloodstream [19,20].

## 3. Mechanism of Spatial Heterogeneity Formation of Intratumoral Microbiota

Intratumoral microbiota refers to microbial communities residing within tumor tissues, including bacteria, fungi, and viruses [2,21]. However, intratumoral microbiota exhibit spatial heterogeneity, which can be reflected by significant differences in microbial composition and abundance across different cancers (Table 1).

For example, in colorectal cancer, *Fusobacterium*, *Bacteroides*, *Campylobacter*, and *Peptostreptococcus* are notably enriched [22,23]. Breast cancer frequently harbors *Methylobacterium radiotolerans*, *Bacillus*, *Enterobacteriaceae*, *Staphylococcus*, and *Staphylococcus epidermidis* [24,25,26]. In pancreatic cancer, species such as *Gammaproteobacteria*, *Pseudoxanthomonas*, *Streptomyces*, *Saccharopolyspora*, and *Bacillus clausii* are dominant [27,28]. Additionally, *Malassezia* species have also been detected in pancreatic cancer [29]. In colorectal cancer, fungi like *Candida*, *Malassezia*, and *Aspergillus* are present and may be associated with immunosuppression [30,31]. Some viruses, including human papillomavirus (HPV) [32], hepatitis B virus (HBV) [33], and herpesviruses [34], are established oncogenic pathogens. However, their classification as part of the “intratumoral microbiota” is still debated. Even within the same tumor, the distribution of intratumoral microbiota varies across internal regions, correlating with hypoxic zones, necrotic areas, or immune cell infiltration regions [35]. The spatial arrangement of intratumoral microbes and their interactions with the host significantly shape the tumor microenvironment, with such heterogeneity closely linked to tumor localization, microenvironmental properties, and host immune dynamics.

**Table 1:** Microbial heterogeneity in different tumors.

Tumor Type	Microorganisms	Abundance Changes	Function
Oral squamous cell carcinoma	*Parvimonas*, *Peptoniphilus*, *Fusobacterium* [22]	Increase	Reduces the protein expression of the tumor suppressor p53
	*Eubacterium infirmum*, *Actinobaculum*, *Selenomas* [36]	Down	Related to immune therapy resistance
Colorectal cancer	*Fusobacterium*, *Bacteroides* [22]	Increase	Activates the MAPK signaling pathway
	*Campylobacter*, *Peptostreptococcus* [23]	Increase	Related to tumor progression
	*Candida* [30]	Increase	Related to tumor progression
	*Malassezia*, *Aspergillus* [31]	Increase	Related to tumor high *KRAS* and MSI mutation
Breast cancer	*Methylobacterium radiotolerans* [24,25]	Increase	Promotes tumor progression
	*Bacillus*, *Enterobacteriaceae*, *Staphylococcus*, *Staphylococcus epidermidis* [26]	Increase	Related to tumor progression
Pancreatic ductal adenocarcinoma	*Gammaproteobacteria* [27]	Increase	Mediates response to gemcitabine-based chemotherapy
	*Pseudoxanthomonas*, *Streptomyces*, *Saccharopolyspora*, *Bacillus clausii* [28]	Increase	Induces an antitumor response and activation of the immune system
	*Malassezia* [29]	Increase	Inhibits tumor growth

Abbreviations: KRAS, Kirsten rat sarcoma viral oncogene homolog; MAPK, mitogen-activated protein kinase; MSI, microsatellite instability.

### 3.1. Tumor Anatomical Location and Local Microenvironment Subsection

Gastrointestinal tumors are directly exposed to the luminal microbiota, resulting in a high similarity between their intratumoral microbiota and gut microbiota [18]. Moreover, numerous oral microbes can colonize tumors through hematogenous dissemination. For instance, *Fusobacterium nucleatum* can reach the breast through the bloodstream, inhibit tumor-infiltrating T cell accumulation, and promote tumor growth and metastasis [20]. The FadA adhesin of *Fusobacterium nucleatum* can bind to E-cadherin on colorectal cancer (CRC) cells, mediating bacterial adhesion, invasion, and colonization [37]. Studies have shown that intratumoral *E. coli* can breach the gut vascular barrier, spread to the liver, and promote pre-metastatic niche formation, driving CRC liver metastasis [18]. Therefore, hematogenous dissemination is crucial both for microbial colonization within tumors and for the distant spread of tumor cells.

**Figure 2:**
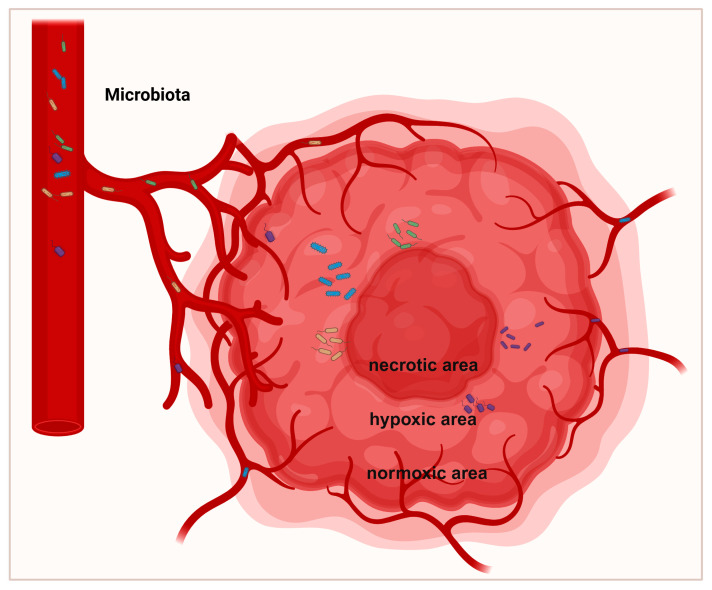
Spatial distribution of intratumoral microbiota. Tumor formation undergoes angiogenesis, resulting in biological changes and adaptive metabolism, leading to the development of defective vessels, hypoxic regions, and heterogeneous tumor cell populations. The defective vascular system permits bacteria to infiltrate tumors. Most bacteria predominantly replicate within hypoxic tumor regions, which are characterized by immunosuppression, nutrient richness, and low oxygen levels.

**Figure 3:**
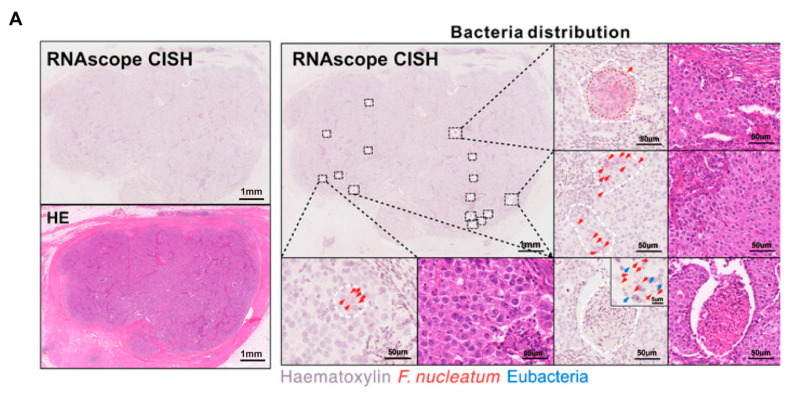

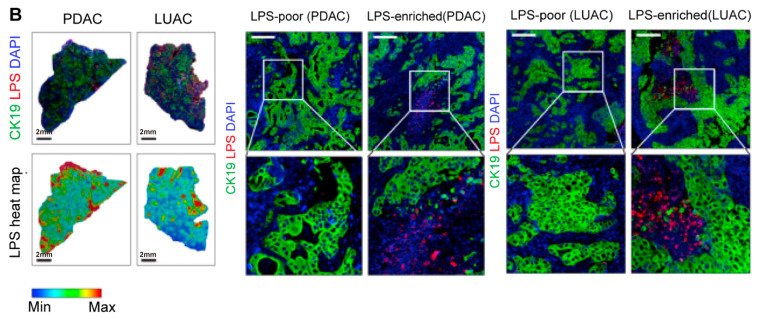
Intratumoral microbiota show uneven spatial distribution within tumors. (**A**) RNAscope FISH and CISH analyses demonstrate the heterogeneous distribution of *F. nucleatum* in breast tumor tissues, characterized by focal clustering in specific tumor regions. The scale bar is shown in the figure, 1 mm and 50 μm [43]. (**B**) Microbial distribution in PDAC and LUAC is spatially heterogeneous. Immunofluorescence (IF) images of CK19 (green), LPS (red), and nuclei (DAPI, blue), and heatmaps of LPS. Scale bars:2 mm (left), 200 μm (right) [48]. Abbreviations: CK19: cytokeratin 19; LPS: lipopolysaccharide; LUAC lung adenocarcinoma; PDAC: pancreatic ductal adenocarcinoma.

The microbial composition varies heterogeneously across different regions within the same tumor. Tumorigenesis undergoes angiogenesis, leading to defective blood vessels, hypoxic regions, and heterogeneous tumor cell populations through biological and metabolic adaptations [38] (Figure 2). *Porphyromonas gingivalis* thrives in the hypoxic microenvironment of PDAC and enhances the tumorigenic activities of pancreatic cancer cells [39]. *Clostridium butyricum* and other nonpathogenic clostridia widely proliferate in hypoxic regions of tumors [40]. *Fusobacterium nucleatum* localizes within hypoxic areas of colorectal cancer and negatively correlates with CD3+ T cell density, contributing to immune suppression [41]. Yamamoto et al. found that in colorectal cancer, *Fusobacterium nucleatum* preferentially accumulates at the invasive tumor front and increases with tumor progression [42]. In breast cancer, *Fusobacterium nucleatum* predominantly localizes in tumor cell-rich areas and promotes cell proliferation and migration via the MAPK signaling pathway [43] (Figure 3). The Warburg effect in tumors increases lactate production, creating an acidic microenvironment that selects for pH-tolerant microbial strains [44]. Hezaveh et al. found that in pancreatic cancer mouse models, tumor-associated macrophage AhR activity relies on intratumoral lactobacilli metabolizing tryptophan into indole, suppressing CD8+ T cell accumulation and correlating with rapid disease progression and mortality [45]. In PDAC, intratumoral bacteria may retrogradely migrate from the duodenum via the pancreatic duct, possibly due to bacterial preference for neutral or slightly alkaline pH environments [46,47].

### 3.2. Bidirectional Regulation of the Immune System

Tumor cells employ multiple mechanisms to evade immune system recognition, leading to insufficient immune activity within tumors and facilitating bacterial colonization [49]. Bacteria are highly organized into microniches associated with immune and epithelial cell functions that promote cancer progression. Galeano Niño et al. reported that bacterial communities mainly reside in microenvironments with poor vascularization, high immunosuppression, and association with malignant cells with low Ki-67 expression [22]. Y. Li et al. applied spatial analysis methods and found that intratumoral microorganisms and T cells co-localize, and this co-dependence is coordinated by T cells, promoting the persistence of microbes within tumors. Furthermore, T cells may enhance tumor vascularization, subsequently favoring microbial enrichment [48]. In addition, bacteria can secrete various immunosuppressive factors to evade immune surveillance and colonize immune-cold regions. For example, the Fap2 protein of *Fusobacterium nucleatum* binds to the inhibitory receptor TIGIT on NK and T cells, thereby inhibiting NK cell-mediated cytotoxicity [50]. Thus, in colorectal adenocarcinoma, despite *Fusobacterium nucleatum* recognizing the overexpressed Gal-GalNAc via Fap2, its distribution through the hematogenous route is still uneven [19]. Moreover, the lipopolysaccharide (LPS) of *Fusobacterium nucleatum* can activate host immune responses through Toll-like receptor 4 (TLR4) [51]. *Fusobacterium nucleatum* can also recruit tumor-infiltrating myeloid-derived suppressor cells (MDSCs) with potent immunosuppressive activity [52]. Additionally, intratumoral microbes may recruit neutrophils, enhancing infiltration and promoting the formation of neutrophil extracellular traps (NETs), and thus capturing and killing some bacteria and causing uneven microbial distribution [53]. T. Zhang et al. found that *Fusobacterium nucleatum* can invade gastric cancer cells and induce the recruitment of tumor-associated neutrophils, thereby altering the tumor immune microenvironment [54].

### 3.3. Interactions Among Microorganisms

During the progression from adenoma to carcinoma, the abundance of intratumoral microbiota also changes. Nakatsu et al. found that in adenomas, Proteobacteria were predominant, and as they were replaced by Firmicutes, the malignancy of tumors increased. Members of Proteobacteria and Firmicutes often exhibit a mutually exclusive relationship in cancers [55]. Consequently, Firmicutes can form a single dominant population. The abundances of *Bacteroides* and *Parvimonas* both increase in colorectal cancer, showing a symbiotic relationship. These microorganisms possess a competitive advantage in colonization, altering the overall microbial profile and thereby modulating the tumor microenvironment. Certain bacteria participate in amino acid metabolism within tumor cells, leading to their localized enrichment. Anaerobic streptococci are involved in glycine fermentation, whereas fermentation of alanine, glutamate, and lysine requires Clostridia [56]. It is likely that the increase in amino acids produced by intratumoral microbes may favor the catabolism of tumor cells, thereby promoting CRC initiation and progression. Interactions between intratumoral fungi and bacteria also influence cancer progression. *Malassezia* has been identified as a potential driver in the progression from adenoma to CRC. The bacteria genus *Streptomyces* shows a positive correlation with *Malassezia*, whereas Eubacterium and Dorea are negatively correlated with *Malassezia* [31].

### 3.4. Effects of Therapeutic Interventions

Meanwhile, the spatial heterogeneity of intratumoral microbiota is influenced by various therapeutic interventions, particularly chemotherapy and antibiotic treatments. Chemotherapeutic agents exert their effects by directly killing tumor cells, but they also significantly impact the tumor microenvironment, particularly by disturbing the intratumoral microbiota [57,58,59]. For example, 5-fluorouracil (5-FU), a commonly used chemotherapeutic agent, can inhibit the growth of *Fusobacterium nucleatum* (Fn) [60]. After 5-FU treatment, microbiota changes are characterized by increased intratumoral microbiota heterogeneity, which may induce immunosuppression and thereby impair therapeutic efficacy [61]. Antibiotics further intensify this effect. Antibiotics, by killing certain bacteria, may shift the tumor microbiota composition [14,62]. Although antibiotic treatment can temporarily reduce the abundance of certain pathogens, its non-selective antimicrobial effects may disturb the entire ecosystem, leading to a loss of beneficial microbiota and consequent dysbiosis [63].

In summary, the spatial heterogeneity of intratumoral microbiota is closely associated with the efficacy of immunotherapy. During treatment, the microbiota composition, metabolic products, and their interactions with immune cells vary across different regions of the tumor microenvironment. These changes may influence differences in treatment responses. Chemotherapy and antibiotic treatments commonly lead to increased spatial heterogeneity of the microbiota. This may promote the enrichment of drug-resistant bacteria and suppress immune function.

## 4. Mechanisms of Spatial Heterogeneity Regulating Immunotherapy

Intratumoral microbiota inhabit the tumor microenvironment (TME), which is highly heterogeneous, with significant regional differences in microbiota distribution, abundance, and function [64,65]. This spatial heterogeneity modulates the response to immunotherapy by influencing multiple aspects, including immune cell infiltration, metabolic microenvironment, immune checkpoint expression, and therapeutic resistance.

### 4.1. Local Regulation of Immune Cell Infiltration by Microbial Spatial Distribution

Recent studies have revealed not only that microbes in the tumor microenvironment (TME) are merely passive residents, but also that their spatial distribution significantly impacts immune cell infiltration and functional states [49]. For instance, commensal *Clostridiales* strains can induce high levels of intratumoral CD8+ T cell infiltration, enhancing tumor immunogenicity [66]. In colorectal cancer (CRC), *Fusobacterium nucleatum* invades tumor tissues and induces the production of inflammatory cytokines (e.g., IL-12, TGF-β, and TNF-α), promoting FOXP3+ CD4 T cell expansion and forming specific spatial co-localization, which is closely associated with poor prognosis [67]. This phenomenon of “spatial co-localization” is not incidental. Studies have shown that tumor-associated microbes locally regulate immune cell recruitment and activation through signaling pathways.

On one hand, microbial components, such as peptidoglycans, LPS, lipopeptides, and flagellin, can activate local immune sensing pathways like TLRs or NOD-like receptors, triggering MyD88-dependent signaling to induce chemokine secretion (e.g., CXCL9, CXCL10, CCL2) and promoting immune cell infiltration, especially T cells, monocytes, and dendritic cells [68,69,70]. On the other hand, intratumoral bacteria can inhabit both tumor cells and immune cells. In melanoma patients, bacterial peptides can be presented by tumor cells, influencing T cell immune responsiveness and reshaping the local immune landscape [71]. In pancreatic ductal adenocarcinoma, increased abundance of three anaerobes, including *Bacteroides*, *Lactobacillus*, and *Peptostreptococcus*, is significantly associated with suppressed effector T cell infiltration and poor prognosis [72]. In lung adenocarcinoma, intratumoral bacteria form immune interaction networks with the host, where increased malignancy is associated with upregulation of γδ T cells and neutrophils, indicating enhanced inflammation and suppressed antitumor immunity, leading to poor overall survival [73].

Moreover, technologies like spatial transcriptomics, spatial FISH imaging, and mass spectrometry have revealed that microbes do not exist in isolation but form “immune-microbiota niches” with local immune cells, playing decisive roles in immune activation or suppression within the TME [43,74]. The Fap2 protein of *Fusobacterium nucleatum* directly interacts with TIGIT, inhibiting NK cell cytotoxicity [50]. Additionally, *F. nucleatum* uses its surface protein CbpF to activate the inhibitory receptor CEACAM1 on T cells, thereby suppressing T cell function and enabling tumor immune evasion [75], providing a novel spatial intervention target for clinical therapy.

### 4.2. Spatial Gradient Regulation of Immune Responses by Microbial Metabolites 

Besides spatial distribution, intratumoral microbiota also regulate the immune system by shaping immune cell functions through spatial gradients of their metabolic products [76]. These metabolic products often establish concentration gradients in the local microenvironment, thereby inducing functional heterogeneity of immune cells across different spatial regions [77]. For example, short-chain fatty acids (SCFAs) like butyrate and propionate are locally produced by anaerobic bacteria in the colon [78]. Intratumoral *F. nucleatum* produces abundant butyrate, which inhibits histone deacetylases (HDACs) in CD8 T cells, induces H3K27 acetylation at the TBX21 promoter, thereby suppressing PD-1, alleviating CD8 T cell exhaustion, and enhancing effector functions [79]. Schulz et al. found in a high-fat diet-induced intestinal cancer model that administration of butyrate could recruit dendritic cells in gut-associated lymphoid tissues, thereby attenuating tumor progression [80]. Butyrate produced by Roseburia can inhibit HDAC2 and increase H3K27 acetylation at the H19 promoter, thereby inducing M2 macrophage polarization and promoting lung cancer metastasis [81].

Moreover, the tryptophan metabolism pathway also plays a crucial role. Intratumoral microbes can induce indoleamine 2,3-dioxygenase (IDO) expression in macrophages, creating a toxic environment with low tryptophan and high kynurenine levels, further impairing peripheral lymphocyte function, inhibiting IFN-γ secretion, and forming an “immune tolerance zone” in tumors or inflamed tissues [50,82].

Finally, lactate, as a co-metabolite of certain bacteria and tumor cells, accumulates locally, acidifies the microenvironment, suppresses effector T cell function, and promotes regulatory T cell development, thereby impeding anti-tumor immune responses [83,84]. However, the impact of lactate on cancer and immune cells within the tumor immune microenvironment can be highly complex. Contrary to lactate’s immunosuppressive effect, Feng et al. demonstrated that sodium lactate enhances the immune protection of stem-like CD8+ T cells during cancer therapy [85]. In summary, the role of metabolic reprogramming within the tumor immune microenvironment in regulating tumor progression is highly complex.

### 4.3. Spatial Regulation of Immune Checkpoint Molecules by Microbes

In the era of immunotherapy, immune checkpoint molecules such as PD-1/PD-L1 and CTLA-4 have become critical targets, and microorganisms can regulate the expression and function of these molecules spatially [86,87]. Programmed cell death protein 1 (PD-1), an immune checkpoint receptor, is abundantly expressed on activated T cells, whereas tumor cells frequently overexpress its ligand, programmed cell death 1 ligand 1 (PD-L1), facilitating immune escape [88]. Immune checkpoint therapies trigger anti-tumor immune responses by blocking the receptor–ligand interactions on T cells and have been applied to treat numerous types of malignancies [89]. PD-1, as the most widely used immune checkpoint inhibitor, plays a pivotal role in immunotherapy research [90]. Intratumoral microorganisms can influence the efficacy of PD-1/PD-L1-mediated anti-tumor immunotherapy [91]. *Fusobacterium nucleatum* can upregulate PD-L1 expression in breast cancer cells and suppress CD8+ T cell-mediated cytotoxicity via the NF-κB/PD-L1 signaling pathway, with expression hotspots typically located at tumor margins coinciding with bacterial aggregates [92]. Intratumoral microbes upregulate tumor cell PD-L1 expression via LPS-mediated TLR4/MyD88 signaling, reshaping the tumor immune microenvironment, with expression hotspots often corresponding to bacterial aggregation sites [93]. Galeano Niño et al. found that in OSCC tumors, the T cell inhibitory receptor PD-1 was overexpressed in bacteria-positive niches, and subsequent RNAscope and IHC analyses confirmed that intratumoral microbes promoted the enrichment of CD11b+ and CD66b+ myeloid cells while inhibiting the accumulation of CD4+ and CD8+ T cells [22]. The spatial variation in expression results in distinct effects of immune checkpoint blockade therapies across tumor regions, possibly accounting for the clinical phenomenon of “partial lesion regression and partial lesion progression” during treatment.

### 4.4. Microbial Spatial Heterogeneity and Immune Therapy Resistance

Immune checkpoint inhibitors (ICIs) have brought revolutionary breakthroughs to the treatment of advanced tumors, yet their response rates remain limited across various solid tumors. Recent studies have shown that the spatial heterogeneity of tumor-associated microorganisms can influence the efficacy of cancer immunotherapy [94]. The distribution of microorganisms within tumor tissues is highly uneven, with spatial heterogeneity manifesting as the enrichment of specific genera in regions such as the tumor core, invasive margins, and immune-exclusion zones [95]. By spatial analysis techniques, Y. Li et al. found that in tumors with low T cell abundance, the microbial composition supports tumor growth, while in tumors rich in T cells, microbes promote B cell infiltration and upregulate immune-stimulatory molecules in intratumoral macrophages, explaining the dual role of microbes in both promoting chemotherapy resistance and tumor progression, and triggering anti-tumor immunity [49]. Moreover, intratumoral microbes can regulate the local metabolic environment, leading to uneven distribution and activity of immunotherapeutic drugs. Geller et al. discovered that *Gammaproteobacteria* can metabolize the chemotherapeutic drug gemcitabine into its inactive form [46]. Therefore, microbial spatial heterogeneity is not only a complex feature of the tumor ecosystem but may also be a key determinant of individual variability in immunotherapy responses. Targeting and recognizing these heterogeneous niches holds promise for overcoming the existing bottlenecks of ICI treatment.

## 5. Immunotherapeutic Potential of Targeting Tumor-Intrinsic Microbial Spatial Heterogeneity

The spatial heterogeneity of intratumoral microbiota can profoundly influence the anti-tumor immune response by reshaping the immune microenvironment, modulating immune cell functions, and altering metabolic interactions [96,97]. Based on this, targeting the spatial heterogeneity of intratumoral microbes has gained increasing clinical application, with emerging strategies such as biomarker development, tumor-targeted engineered bacteria, probiotics, and bacteriophages being explored to enhance cancer immunotherapy efficacy. Currently, multiple clinical trials are focusing on combining microbiome modulation approaches with immunotherapy to overcome therapeutic barriers through microbiota-immune synergy, while also evaluating their safety and clinical translational potential (Table 2).

### 5.1. Biomarker Development

The microbial communities within the tumor microenvironment exhibit significant spatial heterogeneity, which can directly affect immune cell distribution and the nature of immune responses, thereby influencing the efficacy of immunotherapy. For instance, *Fusobacterium nucleatum* has been associated with immunosuppressive environments and drug resistance in colorectal cancer [102], whereas the abundance of certain probiotics correlates positively with the activity of tumor-infiltrating T cells [103]. Characteristics of the tumor-associated microbiome can be utilized to predict the outcomes of immunotherapy. By comparing tumor microbiota profiles before and after immunotherapy, it has been found that spatial heterogeneity of the microbiota is closely related to immune responses [11]. The tumor core is typically an ischemic and hypoxic region, often enriched with immunosuppressive microbes, closely associated with immune evasion characteristics [35]. Therefore, the spatial characteristics of the tumor microbiome can serve as novel predictive biomarkers for forecasting patient responses to immunotherapy.

### 5.2. Engineered Bacteria

The microbiota within the tumor microenvironment (TME) plays a crucial role in immune evasion and resistance mechanisms [104]. The spatial heterogeneity of intratumoral microbiota, particularly in the context of tumor immune evasion, has emerged as a novel area of research. Local delivery of bacteria, especially engineered *Escherichia coli*, to immune-cold tumor regions has become a strategy for remodeling the TME [105]. The *Escherichia coli* Nissle 1917 (EcN) can convert ammonia into L-arginine in the tumor microenvironment, thereby increasing the intratumoral L-arginine concentration. This metabolic intervention significantly enhances T cell infiltration into tumors and acts synergistically with PD-L1 blockade antibodies in antitumor effects [106]. Thus, *Escherichia coli* Nissle 1917 (EcN) has been widely used clinically due to its high safety profile and its selective colonization, survival, and proliferation in hypoxic tumor regions [107,108]. Gurbatri et al. engineered EcN to locally release the cytokine GM-CSF and block PD-L1 and CTLA-4 using nanobodies at the tumor site, resulting in approximately a 50% reduction in tumor burden [99]. Similarly, engineered *Escherichia coli* can colonize tumor areas and induce localized immune responses by expressing specific immunomodulatory factors, such as cytokines or immune regulators [109]. Moreover, “cold tumors” exhibit low T-cell infiltration and immune checkpoint molecule expression, leading to T-cell exhaustion and resistance to immune checkpoint inhibitors in a significant proportion of patients [110]. Engineered *Escherichia coli* can reshape the tumor immune environment by modulating specific microbial communities, thereby overcoming resistance to immunotherapy [111].

### 5.3. Probiotics

Probiotics, as safe and easily applicable microbiota modulators, have the potential to regulate immune responses [112]. For instance, oral administration of Bifidobacterium combined with anti-PD-1 antibody can specifically enhance T-cell responses and dendritic cell (DC) functions, thereby promoting CD8+ T-cell priming and accumulation in the tumor microenvironment and preventing melanoma progression [113]. In addition, probiotics can modulate the efficacy of immunotherapy by influencing tumor-associated gut microbiota [114]. For example, intratumoral *Ruminococcus gnavus* (Rg) and *Blautia producta* (Bp) can degrade lysophosphatidylglycerol, promoting CD8+ T cell activation and maintaining CD8+ T cell immunosurveillance, thereby enhancing antitumor immune responses and controlling colorectal cancer progression [115]. Probiotics can enhance the response to immunotherapy. A clinical trial (NCT03775850) showed that the combination of EDP1503 and pembrolizumab was safe and well tolerated, exerting its effects by enhancing the CD8+ T cell-to-Treg and CD8+ T cell-to-exhausted CD8+ T cell ratios [100]. Another clinical study (NCT05032014) confirmed that probiotic M9 (*Lactobacillus rhamnosus*) combined with PD-1 inhibitors also exerts effects in hepatocellular carcinoma.

Although probiotics hold great promise in tumor immunotherapy, several challenges remain. Issues such as the variability in probiotic strain efficacy, the complexity of the tumor microenvironment, and the long-term safety and tolerability of probiotics require further investigation.

### 5.4. Bacteriophage

Bacteriophage therapy offers an alternative targeted strategy by specifically eliminating certain microbial populations and modulating the microbial composition within the tumor microenvironment, thereby enhancing the efficacy of immunotherapy [116,117]. Studies have shown that *Fusobacterium nucleatum* impairs the efficacy of immunotherapy by promoting the expansion of immunosuppressive T cells and inhibiting effector T cell functions [41]. Dong et al. developed a phage-guided nanodrug by assembling antibacterial silver nanoparticles onto phage surfaces, enabling specific accumulation at tumor sites colonized by *Fusobacterium nucleatum* to exert bactericidal effects. Inhibiting the proliferation of *Fusobacterium nucleatum* effectively suppresses the recruitment of immunosuppressive cells within the colonic tumor microenvironment [101] Additionally, bacteriophage therapy can precisely target pathogenic bacteria within the tumor microenvironment without disturbing normal tissues or microbiota balance, offering high selectivity and low toxicity [118]. Nevertheless, clinical application of phage therapy still faces challenges such as resistance development, targeting efficiency, and optimal timing, all of which require further research and optimization.

In conclusion, phage therapy holds significant immunotherapeutic potential by targeting microbial spatial heterogeneity within the tumor microenvironment and may become a major approach in future cancer treatments.

## 6. Conclusions

The spatial heterogeneity of the tumor microbiome has emerged as a critical and rapidly evolving area in cancer immunotherapy. With the rapid progress of cancer immunotherapy, dynamic changes in the tumor microenvironment (TME) microbiota are believed to play pivotal roles in immune evasion and resistance to immunotherapy [77]. Spatial heterogeneity of the microbiota—referring to the diversity and distribution differences across tumor regions—not only influences immune cell infiltration and function but may also determine immunotherapy outcomes [119].

Although targeting the spatial heterogeneity of tumor-associated microbiota offers new therapeutic potential, it still faces numerous challenges. Primarily, the complexity and heterogeneity of the tumor microbiota make precise localization and targeted interventions difficult. The composition and spatial distribution of tumor microbiota vary significantly across different tumor types and among individual patients. More importantly, current research on the tumor microbiome faces several technical challenges, such as the risk of contamination during sample collection, limited detection sensitivity, and insufficient spatial resolution, all of which may affect the accurate determination of microbial distribution [120,121]. In addition, intervention strategies targeting the tumor microbiome must fully consider potential risks, such as microbial dysbiosis and off-target effects. Therefore, implementing strict aseptic procedures and multimodal detection approaches may help mitigate the aforementioned risks and enhance the reliability of research and its potential for clinical translation [11,122]. Finally, the specific mechanisms by which microbes influence immunotherapy remain to be fully elucidated. Nevertheless, strategies such as engineered bacteria, probiotics, and phage therapy have shown promising preliminary results in preclinical and clinical studies. These therapies not only reshape the microbial landscape within the TME but also enhance the tumor immune milieu by activating immune cells and suppressing immunosuppressive microbes. Furthermore, microbiota modulation can be combined with other immunotherapies, such as immune checkpoint inhibitors (ICIs) and immune cell therapies, to further boost therapeutic efficacy. By integrating microbiology, immunology, and oncology, microbial spatial heterogeneity within tumors is expected to play a crucial role in future cancer therapies, offering more personalized and effective treatment options. With technological innovation, the prospects of cancer immunotherapy will become increasingly promising.

## Figures and Tables

**Table 2:** Clinical trials on intratumoral microbiota modulation and antitumor immunotherapy.

Identifier	Cancer Types	Phase of Trial	Intervention	Primary Outcome Measures	Ref.
**Biomarker**					
NCT03618641	Melanoma	Phase II	TLR9 agonist vidutolimod + anti-PD-1 nivolumab	Major pathologic response (MPR)	[98]
NCT04649476	Oral squamous cell carcinoma	Phase II	Neoadjuvant immunotherapy	Event-free survival (EFS)	[36]
**Engineered Bacteria**					
U1111-1225-7729	Colorectal cancer	Phase I/II	*E. coli* Nissle 1917 (EcN) + PD-L1 inhibitor + CTLA-4 inhibitor	Colonization status of the microbiota	[99]
NCT02718444	Solid neoplasm, lymphoma	Phase I	SYNB1891	ORR	**–**
**Probiotics**					
NCT03775850	Colorectal cancer	Phase I/II	EDP1503 + pembrolizumab	ORR	[100]
NCT05032014	Liver cancer	Phase I/II	Probiotic-M9 + PD-1 inhibitor	ORR	**–**
NCT03829111	Kidney cancer	Phase I	Probiotic CBM588 + PD-1 inhibitor + CTLA-4 inhibitor	ORR	**–**
**Bacteriophage**					
**–**	Colorectal cancer	Pre-clinical	Fn-binding M13 phage + silver nanoparticles (AgNP)	Survival rate	[101]

## Abbreviations

The following abbreviations are used in this manuscript:

AhR	Aryl hydrocarbon receptor
CRC	Colorectal cancer
CK19	Cytokeratin 19
CTLA-4	Cytotoxic T lymphocyte-associated antigen-4
EFS	Event-free survival
Fn	*Fusobacterium nucleatum*
HPV	Human Papillomavirus
HBV	Hepatitis B virus
ICIs	Immune checkpoint inhibitors
KRAS	Kirsten rat sarcoma viral oncogene homolog
LPS	Lipopolysaccharide
LUAC	Lung adenocarcinoma
MAPK	Mitogen-activated protein kinase
MSI	Microsatellite instability
MDSCs	Myeloid-derived suppressor cells
MPR	Major pathologic response
NETs	Neutrophil extracellular traps
ORR	Objective response rate
PDAC	Pancreatic ductal adenocarcinoma
PD-1	Programmed cell death protein 1
PD-L1	Programmed death ligand 1
SCFAs	Short-chain fatty acids
TLR4	Toll-like receptor 4
TME	Tumor microenvironment

## References

[1] de Visser K.E., Joyce J.A. (2023). The Evolving Tumor Microenvironment: From Cancer Initiation to Metastatic Outgrowth. Cancer Cell.

[2] Watkins T.B., Schwarz R.F. (2018). Phylogenetic Quantification of Intratumor Heterogeneity. Cold Spring Harb. Perspect. Med..

[3] Cogdill A.P., Gaudreau P.O., Arora R., Gopalakrishnan V., Wargo J.A. (2018). The Impact of Intratumoral and Gastrointestinal Microbiota on Systemic Cancer Therapy. Trends Immunol..

[4] (2023). Intratumoral Microbiota Contribute to Tumor Heterogeneity and Progression. Cancer Discov..

[5] Giri S., Lamichhane G., Pandey J., Khadayat R., K. C. S., Devkota H.P., Khadka D. (2025). Immune Modulation and Immunotherapy in Solid Tumors: Mechanisms of Resistance and Potential Therapeutic Strategies. Int. J. Mol. Sci..

[6] Matson C., Chervin S., Gajewski T.F. (2021). Cancer and the Microbiome-Influence of the Commensal Microbiota on Cancer, Immune Responses, and Immunotherapy. Gastroenterology.

[7] Mou W., Deng Z., Zhu L., Jiang A., Lin A., Xu L., Deng G., Huang H., Guo Z., Zhu B. (2025). Intratumoral Mycobiome Heterogeneity Influences the Tumor Microenvironment and Immunotherapy Outcomes in Renal Cell Carcinoma. Sci. Adv..

[8] Boesch M., Horvath L., Baty F., Pircher A., Wolf D., Spahn S., Straussman R., Tilg H., Brutsche M.H. (2022). Compartmentalization of the Host Microbiome: How Tumor Microbiota Shapes Checkpoint Immunotherapy Outcome and Offers Therapeutic Prospects. J. Immunother. Cancer.

[9] Li J., Byrne K.T., Yan F., Yamazoe T., Chen Z., Baslan T., Richman L.P., Lin J.H., Sun Y.H., Rech A.J. (2018). Tumor Cell-Intrinsic Factors Underlie Heterogeneity of Immune Cell Infiltration and Response to Immunotherapy. Immunity.

[10] Garrett W.S. (2015). Cancer and the Microbiota. Science.

[11] Nejman D., Livyatan I., Fuks G., Gavert N., Zwang Y., Geller L.T., Rotter-Maskowitz A., Weiser R., Mallel G., Gigi E. (2020). The Human Tumor Microbiome Is Composed of Tumor Type–Specific Intracellular Bacteria. Science.

[12] Wong-Rolle A., Wei H.K., Zhao C., Jin C. (2021). Unexpected Guests in the Tumor Microenvironment: Microbiome in Cancer. Protein Cell.

[13] Urbaniak C., Cummins J., Brackstone M., Macklaim J.M., Gloor G.B., Baban C.K., Scott L., O’Hanlon D.M., Burton J.P., Francis K.P. (2014). Microbiota of Human Breast Tissue. Appl. Environ. Microbiol..

[14] Sethi V., Kurtom S., Tarique M., Lavania S., Malchiodi Z., Hellmund L., Zhang L., Sharma U., Giri B., Garg B. (2018). Gut Microbiota Promotes Tumor Growth in Mice by Modulating Immune Response. Gastroenterology.

[15] Han P., Li L., Liu S., Wang Q., Zhang D., Xu Z., Han P., Li X., Peng Q., Su C. (2022). Receptor Binding and Complex Structures of Human ACE2 to Spike RBD from Omicron and Delta SARS-CoV-2. Cell.

[16] Song X., Wei C., Li X. (2022). The Relationship Between Microbial Community and Breast Cancer. Front. Cell. Infect. Microbiol..

[17] Schorr L., Mathies M., Elinav E., Puschhof J. (2023). Intracellular bacteria in cancer—Prospects and debates. NPJ Biofilms Microbiomes.

[18] Bertocchi A., Carloni S., Ravenda P.S., Bertalot G., Spadoni I., Cascio A.L., Gandini S., Lizier M., Braga D., Asnicar F. (2021). Gut Vascular Barrier Impairment Leads to Intestinal Bacteria Dissemination and Colorectal Cancer Metastasis to Liver. Cancer Cell.

[19] Abed J., Emgård J.E., Zamir G., Faroja M., Almogy G., Grenov A., Sol A., Naor R., Pikarsky E., Atlan K.A. (2016). Fap2 Mediates *Fusobacterium nucleatum* Colorectal Adenocarcinoma Enrichment by Binding to Tumor-Expressed Gal-GalNAc. Cell Host Microbe.

[20] Parhi L., Alon-Maimon T., Sol A., Nejman D., Shhadeh A., Fainsod-Levi T., Yajuk O., Isaacson B., Abed J., Maalouf N. (2020). Breast Cancer Colonization by *Fusobacterium nucleatum* Accelerates Tumor Growth and Metastatic Progression. Nat. Commun..

[21] Narunsky-Haziza L., Sepich-Poore G.D., Livyatan I., Asraf O., Martino C., Nejman D., Gavert N., Stajich J.E., Amit G., González A. (2022). Pan-Cancer Analyses Reveal Cancer-Type-Specific Fungal Ecologies and Bacteriome Interactions. Cell.

[22] Galeano Niño J.L., Wu H., LaCourse K.D., Kempchinsky A.G., Baryiames A., Barber B., Futran N., Houlton J., Sather C., Sicinska E. (2022). Effect of the Intratumoral Microbiota on Spatial and Cellular Heterogeneity in Cancer. Nature.

[23] Darnindro N., Abdullah M., Sukartini N., Rumende C.M., Pitarini A., A Nursyirwan S., Fauzi A., Makmun D., Nelwan E.J., Shatri H. (2025). Differences in Diversity and Composition of Mucosa-Associated Colonic Microbiota in Colorectal Cancer and Non-Colorectal Cancer in Indonesia. World J. Gastroenterol..

[24] Thu M.S., Chotirosniramit K., Nopsopon T., Hirankarn N., Pongpirul K. (2023). Human gut, breast, and oral microbiome in breast cancer: A Systematic Review and Meta-Analysis. Front. Oncol..

[25] Xuan C., Shamonki J.M., Chung A., DiNome M.L., Chung M., Sieling P.A., Lee D.J. (2014). Microbial Dysbiosis Is Associated with Human Breast Cancer. PLoS ONE.

[26] Urbaniak C., Gloor G.B., Brackstone M., Scott L., Tangney M., Reid G. (2016). The Microbiota of Breast Tissue and Its Association with Breast Cancer. Appl. Environ. Microbiol.

[27] May M.S., Park H., Moallem D.H., Seeram D., Dajiang S., Hibshoosh H., Jamison J.K., Uhlemann A.-C., Manji G.A. (2024). Low Bacterial Biomass in Human Pancreatic Cancer and Adjacent Normal Tissue. Int. J. Mol. Sci..

[28] Riquelme E., Zhang Y., Zhang L., Montiel M., Zoltan M., Dong W., Quesada P., Sahin I., Chandra V., Lucas A.S. (2019). Tumor Microbiome Diversity and Composition Influence Pancreatic Cancer Outcomes. Cell.

[29] Aykut B., Pushalkar S., Chen R., Li Q., Abengozar R., Kim J.I., Shadaloey S.A., Wu D., Preiss P., Verma N. (2019). The Fungal Mycobiome Promotes Pancreatic Oncogenesis Via Activation of MBL. Nature.

[30] Zhang Z., Chen Y., Pan X., Li P., Ren Z., Wang X., Chen Y., Shen S., Wang T., Lin A. (2024). IL-1β mediates *Candida tropicalis*-Induced Immunosuppressive Function of MDSCs to Foster Colorectal Cancer. Cell Commun. Signal..

[31] Yuan K., Xu H., Li S., Coker O.O., Liu W., Wang L., Zhang X., Yu J. (2025). Intraneoplastic Fungal Dysbiosis is Associated with Colorectal Cancer Progression and Host Gene Mutation. EBioMedicine.

[32] Zhao G., Chang J., Wei K. (2024). Correlation between breast cancer and human papillomavirus (HPV) infection. Heliyon.

[33] Pinheiro P.S., Zhang J., Setiawan V.W., Cranford H.M., Wong R.J., Liu L. (2025). Liver Cancer Etiology in Asian Subgroups and American Indian, Black, Latino, and White Populations. JAMA Netw. Open.

[34] Chang Y., Cesarman E., Pessin M.S., Lee F., Culpepper J., Knowles D.M., Moore P.S. (1994). Identification of Herpesvirus-like DNA Sequences in Aids-Associated Kaposi’s Sarcoma. Science.

[35] Cummins J., Tangney M. (2013). Bacteria and Tumours: Causative Agents or Opportunistic Inhabitants?. Infect. Agent. Cancer.

[36] Wang X.X., Liu Y.T., Ren J.G., Liu H.M., Fu Q., Yang Y., Fu Q.Y., Chen G. (2024). Salivary Microbiome Relates to Neoadjuvant Immunotherapy Response in OSCC. J. Dent. Res..

[37] Rubinstein M.R., Wang X., Liu W., Hao Y., Cai G., Han Y.W. (2013). *Fusobacterium nucleatum* Promotes Colorectal Carcinogenesis by Modulating E-Cadherin/β-Catenin Signaling via Its Fada Adhesin. Cell Host Microbe.

[38] Wei M.Q., Ellem K.A., Dunn P., West M.J., Bai C.X., Vogelstein B. (2007). Facultative or obligate anaerobic bacteria have the potential for multimodality therapy of solid tumours. Eur. J. Cancer.

[39] Gnanasekaran J., Gallimidi A.B., Saba E., Pandi K., Berchoer L.E., Hermano E., Angabo S., Makkawi H., Khashan A., Daoud A. (2020). Intracellular *Porphyromonas gingivalis* Promotes the Tumorigenic Behavior of Pancreatic Carcinoma Cells. Cancers.

[40] Moese J.R., Moese G. (1964). Oncolysis by Clostridia. I. Activity of *Clostridium butyricum* (M-55) and Other Nonpathogenic Clostridia against the Ehrlich Carcinoma. Cancer Res..

[41] Mima K., Sukawa Y., Nishihara R., Qian Z.R., Yamauchi M., Inamura K., Kim S.A., Masuda A., Nowak J.A., Nosho K. (2015). *Fusobacterium nucleatum* and T Cells in Colorectal Carcinoma. JAMA Oncol..

[42] Yamamoto S., Kinugasa H., Hirai M., Terasawa H., Yasutomi E., Oka S., Ohmori M., Yamasaki Y., Inokuchi T., Harada K. (2021). Heterogeneous Distribution of *Fusobacterium nucleatum* in the Progression of Colorectal Cancer. J. Gastroenterol. Hepatol..

[43] Zhao F., An R., Ma Y., Yu S., Gao Y., Wang Y., Yu H., Xie X., Zhang J. (2025). Integrated spatial multi-omics profiling of *Fusobacterium nucleatum* in breast cancer unveils its role in tumour microenvironment modulation and cancer progression. Clin. Transl. Med..

[44] Hanahan D., Weinberg R.A. (2011). Hallmarks of Cancer: The Next Generation. Cell.

[45] Hezaveh K., Shinde R.S., Klötgen A., Halaby M.J., Lamorte S., Ciudad M.T., Quevedo R., Neufeld L., Liu Z.Q., Jin R. (2022). Tryptophan-derived microbial metabolites activate the aryl hydrocarbon receptor in tumor-associated macrophages to suppress anti-tumor immunity. Immunity.

[46] Geller L.T., Barzily-Rokni M., Danino T., Jonas O.H., Shental N., Nejman D., Gavert N., Zwang Y., Cooper Z.A., Shee K. (2017). Potential role of intratumor bacteria in mediating tumor resistance to the chemotherapeutic drug gemcitabine. Science.

[47] Shirai H., Ito C., Tsukada K. (2022). Ph-Taxis Drives Aerobic Bacteria in Duodenum to Migrate into the Pancreas with Tumors. Sci. Rep..

[48] Li Y., Chang R.B., Stone M.L., Delman D., Markowitz K., Xue Y., Coho H., Herrera V.M., Li J.H., Zhang L. (2024). Multimodal immune phenotyping reveals microbial-T cell interactions that shape pancreatic cancer. Cell Rep. Med..

[49] Bermudes D., Low B., Pawelek J. (2000). Tumor-Targeted Salmonella. Highly Selective Delivery Vectors. Adv. Exp. Med. Biol..

[50] Gur C., Ibrahim Y., Isaacson B., Yamin R., Abed J., Gamliel M., Enk J., Bar-On Y., Stanietsky-Kaynan N., Coppenhagen-Glazer S. (2015). Binding of the Fap2 Protein of *Fusobacterium nucleatum* to Human Inhibitory Receptor TIGIT Protects Tumors from Immune Cell Attack. Immunity.

[51] Chen Y., Peng Y., Yu J., Chen T., Wu Y., Shi L., Li Q., Wu J., Fu X. (2017). Invasive *Fusobacterium nucleatum* activates beta-catenin signaling in colorectal cancer via a TLR4/P-PAK1 cascade. Oncotarget.

[52] Kostic A.D., Chun E., Robertson L., Glickman J.N., Gallini C.A., Michaud M., Clancy T.E., Chung D.C., Lochhead P., Hold G.L. (2013). *Fusobacterium nucleatum* Potentiates Intestinal Tumorigenesis and Modulates the Tumor-Immune Microenvironment. Cell Host Microbe.

[53] Hu M., Hu W., Zhang Z. (2024). Interaction between Intratumoral Microbiota and Neutrophils Influences Tumor Progression. Am. J. Clin. Exp. Immunol..

[54] Zhang T., Li Y., Zhai E., Zhao R., Qian Y., Huang Z., Liu Y., Zhao Z., Xu X., Liu J. (2025). Intratumoral *Fusobacterium nucleatum* Recruits Tumor-Associated Neutrophils to Promote Gastric Cancer Progression and Immune Evasion. Cancer Res..

[55] Nakatsu G., Li X., Zhou H., Sheng J., Wong S.H., Wu W.K.K., Ng S.C., Tsoi H., Dong Y., Zhang N. (2015). Gut mucosal microbiome across stages of colorectal carcinogenesis. Nat. Commun..

[56] Schaechter M. (2009). Encyclopedia of Microbiology.

[57] Penny L.K., Wallace H.M. (2015). The Challenges for Cancer Chemoprevention. Chem. Soc. Rev..

[58] Wang M., Rousseau B., Qiu K., Huang G., Zhang Y., Su H., Le Bihan-Benjamin C., Khati I., Artz O., Foote M.B. (2023). Killing tumor-associated bacteria with a liposomal antibiotic generates neoantigens that induce anti-tumor immune responses. Nat. Biotechnol..

[59] Xin Y.-T., Wang L.-Y., Chang H.-H., Ma F.-H., Sun M.-L., Chen L., Gao H. (2022). Construction of PAMAM-based Nanocomplex Conjugated with Pt(IV)-complex and Lauric Acid Exerting Both Anti-tumor and Antibacterial Effects. Chin. J. Polym. Sci..

[60] LaCourse K.D., Zepeda-Rivera M., Kempchinsky A.G., Baryiames A., Minot S.S., Johnston C.D., Bullman S. (2022). The cancer chemotherapeutic 5-fluorouracil is a potent *Fusobacterium nucleatum* inhibitor and its activity is modified by intratumoral microbiota. Cell Rep..

[61] Mima K., Nishihara R., Qian Z.R., Cao Y., Sukawa Y., Nowak J.A., Yang J., Dou R., Masugi Y., Song M. (2016). *Fusobacterium nucleatum* in Colo-rectal Carcinoma Tissue and Patient Prognosis. Gut.

[62] Riquelme E., Maitra A., McAllister F. (2018). Immunotherapy for Pancreatic Cancer: More Than Just a Gut Feeling. Cancer Discov..

[63] Fan D., A Coughlin L., Neubauer M.M., Kim J., Kim M.S., Zhan X., Simms-Waldrip T.R., Xie Y., Hooper L.V., Koh A.Y. (2015). Activation of HIF-1α and LL-37 by commensal bacteria inhibits *Candida albicans* colonization. Nat. Med..

[64] Liu S., Pan Y., Zheng C., Zheng Q., Du Y., Zheng Y., Tang H., Liu X., Mou J., Zeng X. (2025). Tumor-colonizing *Pseudoalteromonas elyakovii* metabolically reprograms the tumor microenvironment and promotes breast ductal carcinoma. mBio.

[65] Ying C., Wu J., Cai K., Xiao X., Chen Y., Zhang X., Deng S., Pei C., Chen Y., Xie Z. (2025). Bifidobacterium Longum Subsp. Longum Xz01 Delays the Progression of Colon Cancer in Mice through the Interaction between the Microbial Spatial Distribution and Tumour Immunity. Int. Immunopharmacol..

[66] Montalban-Arques A., Katkeviciute E., Busenhart P., Bircher A., Wirbel J., Zeller G., Morsy Y., Borsig L., Garzon J.F.G., Müller A. (2021). Commensal *Clostridiales* strains mediate effective anti-cancer immune response against solid tumors. Cell Host Microbe.

[67] Saito T., Nishikawa H., Wada H., Nagano Y., Sugiyama D., Atarashi K., Maeda Y., Hamaguchi M., Ohkura N., Sato E. (2016). Two FOXP3^+^CD4^+^ T cell subpopulations distinctly control the prognosis of colorectal cancers. Nat. Med..

[68] Coussens L.M., Zitvogel L., Palucka A.K. (2013). Neutralizing Tumor-Promoting Chronic Inflammation: A Magic Bullet?. Science.

[69] Zitvogel L., Ayyoub M., Routy B., Kroemer G. (2016). Microbiome and Anticancer Immunosurveillance. Cell.

[70] Liu D., Zhu Y., Hou Z., Wang H., Li Q. (2025). Polysaccharides from *Astragalus membranaceus* Bunge Alleviate LPS-Induced Neuroinflammation in Mice by Modulating Microbe-Metabolite-Brain Axis and MAPK/NF-κB Signaling Pathway. Int. J. Biol. Macromol..

[71] Kalaora S., Nagler A., Nejman D., Alon M., Barbolin C., Barnea E., Ketelaars S.L.C., Cheng K., Vervier K., Shental N. (2021). Identification of Bacteria-Derived HLA-Bound Peptides in Melanoma. Nature.

[72] Abe S., Masuda A., Matsumoto T., Inoue J., Toyama H., Sakai A., Kobayashi T., Tanaka T., Tsujimae M., Yamakawa K. (2024). Impact of intratumoral microbiome on tumor immunity and prognosis in human pancreatic ductal adenocarcinoma. J. Gastroenterol..

[73] Liang P., Chen Q., Chen X., Zhang X., Xiao Y., Liang G., Liu M., He J., Liang W., Liang Y. (2024). Microbiota modulate immune repertories in lung adenocarcinoma via microbiota-immunity interactive network. Transl. Lung Cancer Res..

[74] Li T., Zhao Z., Peng M., Zhang L., Wang C., Luo F., Zeng M., Sun K., Fang Z., Luo Y. (2025). Multi-omics analysis reveals the interplay between intratumoral bacteria and glioma. mSystems.

[75] Galaski J., Shhadeh A., Umaña A., Yoo C.C., Arpinati L., Isaacson B., Berhani O., Singer B.B., Slade D.J., Bachrach G. (2021). *Fusobacterium nucleatum* CbpF Mediates Inhibition of T Cell Function Through CEACAM1 Activation. Front. Cell. Infect. Microbiol..

[76] Carmona-Fontaine C., Deforet M., Akkari L., Thompson C.B., Joyce J.A., Xavier J.B. (2017). Metabolic origins of spatial organization in the tumor microenvironment. Proc. Natl. Acad. Sci. USA.

[77] Fu T., Dai L.-J., Wu S.-Y., Xiao Y., Ma D., Jiang Y.-Z., Shao Z.-M. (2021). Spatial architecture of the immune microenvironment orchestrates tumor immunity and therapeutic response. J. Hematol. Oncol..

[78] Topping D.L. (1996). Short-chain fatty acids produced by intestinal bacteria. Asia Pac. J. Clin. Nutr..

[79] Wang X., Fang Y., Liang W., Wong C.C., Qin H., Gao Y., Liang M., Song L., Zhang Y., Fan M. (2025). *Fusobacterium nucleatum* facilitates anti-PD-1 therapy in microsatellite stable colorectal cancer. Cancer Cell.

[80] Schulz M.D., Atay C., Heringer J., Romrig F.K., Schwitalla S., Aydin B., Ziegler P.K., Varga J., Reindl W., Pommerenke C. (2014). High-fat-diet-mediated dysbiosis promotes intestinal carcinogenesis independently of obesity. Nature.

[81] Ma Y., Chen H., Li H., Zheng M., Zuo X., Wang W., Wang S., Lu Y., Wang J., Li Y. (2024). Intratumor Microbiome-Derived Butyrate Promotes Lung Cancer Metastasis. Cell Rep. Med..

[82] Xue Y., Xiao H., Guo S., Xu B., Liao Y., Wu Y., Zhang G. (2018). Indoleamine 2,3-dioxygenase expression regulates the survival and proliferation of *Fusobacterium nucleatum* in THP-1-derived macrophages. Cell Death Dis..

[83] Hayes C., Donohoe C.L., Davern M., Donlon N.E. (2021). The oncogenic and clinical implications of lactate induced immunosuppression in the tumour microenvironment. Cancer Lett..

[84] Liu Y., Cao L. (2024). Intratumoral *Lactobacillus iners* as a poor prognostic biomarker and potential therapeutic target for cervical cancer. Front. Cell. Infect. Microbiol..

[85] Feng Q., Liu Z., Yu X., Huang T., Chen J., Wang J., Wilhelm J., Li S., Song J., Li W. (2022). Lactate increases stemness of CD8+ T cells to augment anti-tumor immunity. Nat. Commun..

[86] Weng Y., Wang L., Wang Y., Xu J., Fan X., Luo S., Hua Q., Xu J., Liu G., Zhao K.-B. (2025). Spatial Organization of Macrophages in CTL-Rich Hepatocellular Carcinoma Influences CTL Antitumor Activity. Cancer Immunol. Res..

[87] Machuca-Ostos M., de Martines T., Yoshimura K., Mitsuda J., Saburi S., Kimura A., Morimoto H., Yoshizawa K., Sakurai N., Murakami N. (2025). Applications of Multiplex Immunohistochemistry in Evaluating Spatiotemporal Heterogeneity of T Cells. Immuno.

[88] Okazaki T., Honjo T. (2006). The PD-1–PD-L pathway in immunological tolerance. Trends Immunol..

[89] Couzin-Frankel J. (2013). Breakthrough of the Year 2013. Cancer Immunotherapy. Science.

[90] Freeman G.J., Long A.J., Iwai Y., Bourque K., Chernova T., Nishimura H., Fitz L.J., Malenkovich N., Okazaki T., Byrne M.C. (2000). Engagement of the PD-1 Immunoinhibitory Receptor by a Novel B7 Family Member Leads to Negative Regulation of Lymphocyte Activation. J. Exp. Med..

[91] Yi M., Zheng X., Niu M., Zhu S., Ge H., Wu K. (2022). Combination strategies with PD-1/PD-L1 blockade: Current advances and future directions. Mol. Cancer.

[92] Guo J., Zhu P., Li J., Xu L., Tang Y., Liu X., Guo S., Xia J. (2024). *Fusobacterium nucleatum* promotes PD-L1 expression in cancer cells to evade CD8+ T cell killing in breast cancer. Hum. Immunol..

[93] Yin H., Pu N., Chen Q., Zhang J., Zhao G., Xu X., Wang D., Kuang T., Jin D., Lou W. (2021). Gut-Derived Lipopolysaccharide Remodels Tumoral Microenvironment and Synergizes with PD-L1 Checkpoint Blockade via TLR4/MyD88/AKT/NF-κB Pathway in Pancreatic Cancer. Cell Death Dis..

[94] Toley B.J., Forbes N.S. (2011). Motility is critical for effective distribution and accumulation of bacteria in tumor tissue. Integr. Biol..

[95] Yang L., Li A., Wang Y., Zhang Y. (2023). Intratumoral Microbiota: Roles in Cancer Initiation, Development and Therapeutic Efficacy. Signal Transduct. Target. Ther..

[96] Walsh L.A., Quail D.F. (2023). Decoding the Tumor Microenvironment with Spatial Technologies. Nat. Immunol..

[97] Hu J., Coleman K., Zhang D., Lee E.B., Kadara H., Wang L., Li M. (2023). Deciphering Tumor Ecosystems at Super Resolution from Spatial Transcriptomics with Tesla. Cell Syst..

[98] Davar D., Morrison R.M., Dzutsev A.K., Karunamurthy A., Chauvin J.-M., Amatore F., Deutsch J.S., Das Neves R.X., Rodrigues R.R., McCulloch J.A. (2024). Neoadjuvant vidutolimod and nivolumab in high-risk resectable melanoma: A prospective phase II trial. Cancer Cell.

[99] Gurbatri C.R., Radford G.A., Vrbanac L., Im J., Thomas E.M., Coker C., Taylor S.R., Jang Y., Sivan A., Rhee K. (2024). Engineering tumor-colonizing *E. coli* Nissle 1917 for detection and treatment of colorectal neoplasia. Nat. Commun..

[100] McHale D., Francisco-Anderson L., Sandy P., Shariffudin S., Goldberg M., Gardner H., Abdou M., Kashyap S., Argueta S., Parameswaran P. (2020). P-325 Oral delivery of a single microbial strain, EDP1503, induces anti-tumor responses via gut-mediated activation of both innate and adaptive immunity. Ann. Oncol..

[101] Dong X., Pan P., Zheng D.-W., Bao P., Zeng X., Zhang X.-Z. (2020). Bioinorganic hybrid bacteriophage for modulation of intestinal microbiota to remodel tumor-immune microenvironment against colorectal cancer. Sci. Adv..

[102] Wu J., Li Q., Fu X. (2019). *Fusobacterium nucleatum* Contributes to the Carcinogenesis of Colorectal Cancer by Inducing Inflammation and Suppressing Host Immunity. Transl. Oncol..

[103] Shida K., Nanno M., Nagata S. (2011). Flexible cytokine production by macrophages and T cells in response to probiotic bacteria: A possible mechanism by which probiotics exert multifunctional immune regulatory activities. Gut Microbes.

[104] Liu X., Sun M., Pu F., Ren J., Qu X. (2023). Transforming Intratumor Bacteria into Immunopotentiators to Reverse Cold Tumors for Enhanced Immuno-Chemodynamic Therapy of Triple-Negative Breast Cancer. J. Am. Chem. Soc..

[105] Gurbatri C.R., Arpaia N., Danino T. (2022). Engineering bacteria as interactive cancer therapies. Science.

[106] Canale F.P., Basso C., Antonini G., Perotti M., Li N., Sokolovska A., Neumann J., James M.J., Geiger S., Jin W. (2021). Metabolic Modulation of Tumours with Engineered Bacteria for Immunotherapy. Nature.

[107] Pan J., Li H., Jin K., Jiang H., Li K., Tang Y., Liu Z., Zhang K., Chen K., Xu Z. (2022). Periosteal topology creates an osteo-friendly microenvironment for progenitor cells. Mater. Today Bio.

[108] Ikebe H., Nakao K., Hisamura E., Furukori M., Nakayama Y., Hosokai T., Yang M., Liu G., Yasuda T., Albrecht K. (2023). Thermally activated delayed fluorescence carbazole-triazine dendrimer with bulky substituents. Aggregate.

[109] Zhou S., Gravekamp C., Bermudes D., Liu K. (2018). Tumour-targeting bacteria engineered to fight cancer. Nat. Rev. Cancer.

[110] Li Y., Xiao K., Huang C., Wang J., Gao M., Hu A., Tang Q., Fan B., Xu Y., Chen X. (2020). Enhanced Potassium-Ion Storage of the 3D Carbon Superstructure by Manipulating the Nitrogen-Doped Species and Morphology. Nano-Micro Lett..

[111] Medzhitov R. (2008). Origin and physiological roles of inflammation. Nature.

[112] Hill C., Guarner F., Reid G., Gibson G.R., Merenstein D.J., Pot B., Morelli L., Canani R.B., Flint H.J., Salminen S. (2014). Expert consensus document: The International Scientific Association for Probiotics and Prebiotics consensus statement on the scope and appropriate use of the term probiotic. Nat. Rev. Gastroenterol. Hepatol..

[113] Matson V., Fessler J., Bao R., Chongsuwat T., Zha Y.Y., Alegr M.L., Luke J.J., Gajewski T.F. (2018). The Commensal Microbiome Is Associated with Anti-PD-1 Efficacy in Metastatic Melanoma Patients. Science.

[114] Sivan A., Corrales L., Hubert N., Williams J.B., Aquino-Michaels K., Earley Z.M., Benyamin F.W., Lei Y.M., Jabri B., Alegre M.L. (2015). Commensal *Bifidobacterium* Promotes Antitumor Immunity and Facilitates Anti-PD-L1 Efficacy. Science.

[115] Zhang X., Yu D., Wu D., Gao X., Shao F., Zhao M., Wang J., Ma J., Wang W., Qin X. (2023). Tissue-resident Lachnospiraceae family bacteria protect against colorectal carcinogenesis by promoting tumor immune surveillance. Cell Host Microbe.

[116] Zheng D.-W., Dong X., Pan P., Chen K.-W., Fan J.-X., Cheng S.-X., Zhang X.-Z. (2019). Phage-guided modulation of the gut microbiota of mouse models of colorectal cancer augments their responses to chemotherapy. Nat. Biomed. Eng..

[117] Kabwe M., Dashper S., Bachrach G., Tucci J. (2021). Bacteriophage Manipulation of the Microbiome Associated with Tumour Microenvironments—Can This Improve Cancer Therapeutic Response?. FEMS Microbiol. Rev..

[118] Ganeshan S.D., Hosseinidoust Z. (2019). Phage Therapy with a Focus on the Human Microbiota. Antibiotics.

[119] Tsujikawa T., Mitsuda J., Ogi H., Miyagawa-Hayashino A., Konishi E., Itoh K., Hirano S. (2020). Prognostic Significance of Spatial Immune Profiles in Human Solid Cancers. Cancer Sci..

[120] Knight R., Vrbanac A., Taylor B.C., Aksenov A., Callewaert C., Debelius J., Gonzalez A., Kosciolek T., McCall L.-I., McDonald D. (2018). Best practices for analysing microbiomes. Nat. Rev. Microbiol..

[121] Eisenhofer R., Minich J.J., Marotz C., Cooper A., Knight R., Weyrich L.S. (2019). Contamination in Low Microbial Biomass Microbiome Studies: Issues and Recommendations. Trends Microbiol..

[122] Kuchina A., Brettner L.M., Paleologu L., Roco C.M., Rosenberg A.B., Carignano A., Kibler R., Hirano M., DePaolo R.W., Seelig G. (2021). Microbial single-cell RNA sequencing by split-pool barcoding. Science.

